# The Neural Signature of Statistical Learning of Orthography

**DOI:** 10.3389/fnhum.2020.00026

**Published:** 2020-03-05

**Authors:** Xiuhong Tong, Yi Wang, Shelley Xiuli Tong

**Affiliations:** ^1^Department of Psychology, The Education University of Hong Kong, Tai Po, Hong Kong; ^2^Institute of Psychological Sciences, Hangzhou Normal University, Hangzhou, China; ^3^Human Communication, Development, and Information Sciences, Faculty of Education, The University of Hong Kong, Pokfulam, Hong Kong

**Keywords:** event-related potentials, statistical learning, orthographic learning, Chinese, N1

## Abstract

While an increasing number of behavioral studies suggest the importance of statistical learning in acquiring orthographic regularity across writing systems, no direct neural evidence supports this claim. The present study used event-related potentials (ERPs) to investigate the time course and the neural correlate of statistical learning of positional consistency in Chinese orthography. Visual ERPs were recorded, while Chinese adults performed an orthographic statistical learning task involving artificial characters varying in high, moderate, and low levels of positional consistency. The negative ERP deflection at the N1 time window, typically linked with orthographic regularity processing, was found in orthographic statistical learning with the low and moderate consistencies eliciting larger neural responses than the high consistency in the time window of 150–210 ms over occipital–temporal brain areas. These results suggest that orthographic statistical learning begins within the first 210 ms and that the N1 might be its neural indicator.

## Introduction

An increasing number of recent studies show that statistical learning is not only useful for spoken language acquisition (for a review, see Erickson and Thiessen, 2015) but also plays a role in orthographic learning, which is the process of acquiring word-specific orthographic representations essential for reading and writing (for a review, see Castles et al., 2018). In particular, statistical learning, or the ability to extract and integrate statistical properties of environmental input, such as frequency and variability, has been shown to be a powerful tool that helps Chinese children learn a large number of visually complex characters in the process of becoming literate (e.g., Arciuli and Simpson, 2011; Yin and McBride, 2015; He and Tong, 2017). However, as statistical learning has only been investigated behaviorally in these previous studies, the neural mechanism of statistical learning of orthographic regularities remains unexplored. Thus, in the present study, we employ event-related potentials (ERPs) to examine the electrophysiological correlate of statistical learning of positional consistency of radicals, a key aspect of Chinese character orthography.

Since over 80% of Chinese characters are semantic–phonetic compound characters that comprise semantic and phonetic radicals, the positional consistency of these radicals is a critical statistical property that can be used to identify the legal positions of radicals (Shu et al., 2003). The positional consistency of these radicals indicates how frequently a radical occurs in a given location within characters, and it can vary from 0% to 100%. That is, some radicals only appear in a specific location when forming a Chinese character. For example, the radicals 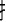, 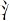, 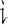, 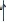, 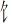, and 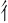 always appear on the left, and never in any other position. Thus, the positional consistency for these radicals on the left is 100%, while it is 0% for the other locations. However, not all radicals follow an all (100%) or none (0%) consistency regularity. Most radicals appear in different positions when combined with other components to form characters. For example, the radical 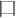 can appear on the left (e.g., 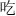, 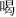, 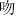, 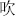, 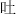, 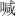, 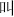), right (e.g., 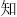, 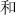), top (e.g., 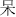, 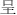), bottom (e.g., 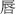, 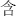, 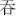, 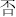, 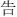), or inside (e.g., 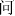). Thus, the positional consistency for the radical 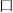 varies across different characters. The existence of this distributional information of a radical’s position raises two questions: (1) Does the statistical information of a radical’s position become encoded during the learning process? and (2) What electrophysiological indicator is linked with this process?

In fact, a few studies have demonstrated that young Chinese children are able to extract the statistical distributional information of radicals (e.g., Tong and McBride, 2014; Yin and McBride, 2015; He and Tong, 2017) and use that information in their subsequent character recognition and encoding (e.g., He and Tong, 2017). For example, Tong and McBride (2014) used an orthographic regularity elicitation paradigm in which participants were asked to invent novel characters using untaught structural units of characters (i.e., stroke patterns) and demonstrated that even preschool children were sensitive to the positional constraints of structural components of characters and that their ability to use the positional constraints of stroke patterns improved as their reading experience increased. Furthermore, He and Tong (2017) employed a modified classical statistical learning paradigm, i.e., artificial orthography learning, in which a set of Chinese-like logographic characters was created using an ideographic script (i.e., Dongba) and a syllabic script (i.e., Geba). After a short exposure to a subset of novel logographic characters, school-aged children were able to distinguish characters containing radicals in legal positions from those containing radicals in illegal positions (He and Tong, 2017).

However, these previous behavioral studies primarily make use of reaction time or response accuracy as indices of statistical learning. These measures are often indirect and rarely straightforward since they may result from a compound function of perception, cognition, attention, and motor control (e.g., Daltrozzo et al., 2017). Thus, variations in reaction time and accuracy may be difficult to attribute to variations in a specific cognitive process (Landi and Perfetti, 2007), such as statistical learning. In the present study, by relying on electrophysiological, rather than behavioral, responses to stimuli, we examine the neural processing of statistical learning of positional consistency of radicals, both in terms of robustness and speed of the responses. Also, since the ERP approach provides a continuous recording of brain activity with millisecond temporal resolution (Molfese et al., 1999), and since ERPs are time locked to the onset of stimuli, we were able to disentangle statistical learning processes that cannot be predicted from behavioral data alone, such as when statistical learning occurs (Howard-Jones et al., 2016).

To date, several studies have employed the ERP approach to investigate the electrophysiological correlates of statistical learning in speech or artificial grammar learning (for a review see Daltrozzo and Conway, 2014). For example, Kooijman et al. (2005) found that a N400-like component was elicited when 10-months-old prelinguistic infants were sensitive to the boundary of words in continuous speech. Also, Teinonen et al. (2009) showed that even sleeping newborn infants are able to use statistical patterns of speech input to detect the word boundaries in a continuous stream of syllables containing no morphological cues. This sensitivity was reflected by a late negativity in the N400 time window of 340–390 ms (Teinonen et al., 2009). Similarly, an ERP study in adults revealed that the initial syllable of pseudowords elicited a larger N100 than the medial and final syllables for both before and after training phases in fast learners, but not in slow learners, with a similar effect occurring in the N400 component (Sanders et al., 2002).

These ERP studies on statistical learning in language acquisition and processing motivated us to examine the neural mechanism of statistical learning of orthographic regularities in Chinese. Specifically, we examined the time course and neural correlate of statistical learning of positional consistency in Chinese adult learners. This would allow us to examine where and when orthographic statistical learning occurs. The statistical property was manipulated by varying the consistency levels (i.e., high, moderate, and low) of target radicals embedded in artificial pseudocharacters. Although no empirical studies have directly examined the neural process of statistical learning of orthographic regularities, there have been studies focusing on the consistency effect in Chinese visual word recognition. For example, Lee et al. (2007) used the ERP technique to investigate the neural locus of the consistency effect of phonetic radicals of Chinese characters in a homophone judgment task. The authors reported that the low-consistency characters produced a greater N170 amplitude in the temporal–occipital region and a greater P200 amplitude in the frontal region than the high-consistency characters, and that high-consistency characters elicited a greater N400 amplitude than low-consistency characters. The N170 and N400 are two different ERP components that associate with different information process during word recognition. The N170 is a negative-going ERP component peaking at around 200 ms after stimulus onset with localization over the left occipital–temporal cortex in skilled readers (e.g., Rossion et al., 2002; Maurer et al., 2008; Tong and McBride, 2018). The N170 is found to associate with orthographic processing or visual word form analysis. In contrast, the N400 component is a negative component that is related to semantic and expectancy of a given word to end a sentence (Kutas and Hillyard, 1980) or reflects a later stage of lexical processing (Lee et al., 2007). Different from previous studies, the present study mainly focused on orthographic learning of the positional regularity rather than on how the positional consistency influences visual word recognition. In addition, different tasks may determine the degree of top–down semantic information processing involved in visual word recognition (Maurer et al., 2005; Eberhard-Moscicka et al., 2015). For example, in the repetition detection task used in the present study, the participants were required to make responses only to nontarget stimuli based on a visual analysis of presented stimuli; in this way, the top–down influences from semantics could be minimized. Thus, we expect that the statistical learning of positional regularities of radicals would occur in an early time window with topographic distribution over the posterior brain areas (i.e., N170 time window).

## Materials and Methods

### Participants

We recruited 29 Chinese speaking undergraduate or graduate students aged between 18 and 26 years from a local university to participate in the experiment. Two participants’ ERP data had excessive artifacts and two participants’ accuracy in the learning phase was lower than 50%. They were excluded from analysis. All the 25 participants (eight males) included in the analysis were Chinese Mandarin native speakers and had never performed similar experiments before. All the participants were right-handed and had normal or corrected-to-normal vision. Sixty Yuan (approximately 9 U.S. dollars) was given to each participant to express our gratuity to their participation.

### Materials and Design

The core learning stimuli were 30 pseudocharacters adopted and modified from a recent study by He and Tong (2017). These pseudocharacters were created using real Geba and Dongba characters once used by the Naxi minority in Western China (Li, 2014) but which have never been exposed or taught to the participants.

The 30 pseudocharacters were created by combining six target radicals with five control radicals. The control radicals carried no positional preference among items. However, the target radicals were manipulated to carry different positional consistencies. In six target radicals that the participants had to learn, three target radicals appeared in one position (i.e., top), whereas the other three appeared in the opposite position (i.e., bottom). The target radicals for each assigned position varied in consistency: high (100%), moderate (80%), and low (60%). These levels were selected according to the statistical properties of the Chinese characters. For example, to create 100% consistency, the target radical 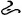 appeared on the top in all five pseudocharacters. At 80% consistency, the radical 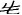 appeared on the top in four pseudocharacters and on the bottom in one pseudocharacter. At 60% consistency, the radical 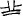 appeared on the top in three pseudocharacters and on the bottom in two pseudocharacters.

### Procedure

**Figure 1:**
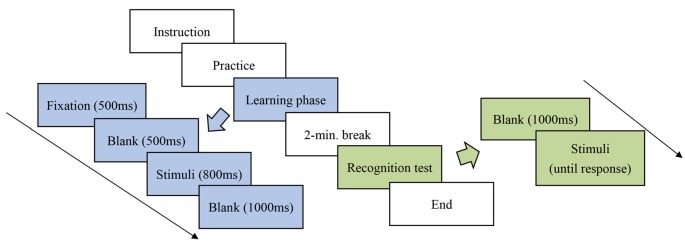
Procedure for the learning and recognition tests.

All the participants were individually tested in a sound-attenuated electroencephalographic (EEG) lab at the university. The participants had to complete both parts of the experiment comprised of a learning phase and a recognition test with a 2-min break in between. The participants’ neural activity was recorded only during the learning phase. The participants’ response accuracy and reaction time, however, were recorded during both phases. The procedure for the learning and recognition tests is illustrated in Figure 1.

In the learning phase, a revised statistical learning paradigm, which has been used successfully in prior studies (He and Tong, 2017), was adopted to assess the participants’ statistical learning process. The stimuli were shown on the monitor using E-Prime 2.0 software (Psychology Software Tools, Pittsburgh, PA, USA). The participants were exposed to each continuous sequence of pseudocharacters in a fixed pseudo-randomized order at the center of the monitor. Each stimulus was repeated 24 times in the learning phase, with a total of 720 trials. At the beginning of each trial, a fixation “+” lasting 500 ms appeared on the monitor screen. Next, a blank screen appeared for 500 ms, followed by a pseudocharacter with a duration of 800 ms. After each stimulus presentation, a blank screen was shown for 1,000 ms as an interstimulus interval (ISI). The participants were asked to press the SPACEBAR key whenever two identical stimuli were presented continuously. Ten practice trials were administered in order to familiarize the participants with the experiment.

In the recognition test, the participants were shown 30 stimuli, half of which had appeared in the learning phase, while the other half were novel stimuli serving as foils by reversing the positions of the target and control radicals. The participants were required to identify if they had seen the stimuli in the learning phase by pressing corresponding keyboard buttons. The stimuli remained visible until the participants pressed the key.

### EEG Recordings and Data Analysis

We used the Brain Product 32-channel Ag/AgCl system (Brain Products Inc) to record the participants’ EEG activity. The EEG was recorded continuously at a sampling rate of 500 Hz with the FCz electrode as the online reference. Electrode impedances were kept below 5 kΩ. The EEG data were preprocessed with EEGLAB (Delorme and Makeig, 2004). Continuous EEG data were filtered with 0.05 Hz to 100 Hz as the online band-pass filter. The filtered data were segmented into epochs from −100 ms to 600 ms and time locked to the target stimuli with an offline band-pass filter of 0.2 Hz to 30 Hz. The ICA approach was applied to reject eye blinks. According to the average reference, the data were corrected to a −100- to 0-ms baseline. The ERPs were averaged within each condition.

Statistical analyses were performed on the amplitude measured as the mean amplitude across the 150- to 210-ms time window. We used the mean amplitude, rather than the peak amplitude, and latency to index the ERP components because the mean amplitude approach has many advantages over the peak amplitude approach (Luck, 2014). For example, the mean amplitude approach is suggested to be more reliable than the peak amplitude approach because peak amplitude is easily influenced by noise. In contrast, the mean amplitude filters out noise at high and intermediate frequencies (Luck, 2014). In addition, peak occurs at different times at different electrode sites, which is against the hypothesis that an ERP component in the brain has the same timing at every electrode side. Furthermore, the mean amplitude is insensitive to trail-to-trial latency variability; in contrast, the peak amplitude is strongly influenced by trial-to-trial latency variability (Luck, 2014). Four electrodes (i.e., P7, O1, P8, and O2) were selected for statistical analyses on the topographic map in the most negative field over both hemispheres across experimental conditions. Repeated measures analysis of variance (ANOVA), with experimental conditions (low-, moderate-, and high-consistency levels), hemispheres (left, right), and electrodes (P7, O1, P8, and O2) as within-subjects factors, were performed in the selected time window. The Greenhouse–Geisser adjustment to the degrees of freedom was used to correct for violations of sphericity associated with a repeated measure.

## Results

### Behavioral Data

As shown in Table 1, for the learning test, the participants’ response accuracy ranged from 68.3% to 100% with a mean of 88.3%, suggesting that all the participants attended to the stimuli during the learning phase, and all the participants were retained for the analysis of recognition test performance.

**Table 1:** Reaction time (ms) and accuracy rate in the learning and recognition phases.

Conditions	Learning phase		Recognition phase	
	Reaction time	Accuracy rate	Reaction time	Accuracy rate
Low consistency	670.184 (115.297)	0.887 (0.091)	1,345.247 (473.403)	0.612 (0.120)
Moderate consistency	671.308 (113.925)	0.886 (0.079)	1,480.098 (956.241)	0.644 (0.133)
High consistency	669.105 (112.214)	0.875 (0.099)	1,464.884 (566.967)	0.852 (0.105)

*Note. The numbers in parentheses are standard deviations*.

For the recognition test, the participants’ overall mean recognition accuracy (mean = 70.21%) was significantly higher than 50% chance (*t*_(24)_ = 13.48, *p* < 0.001). One-sample *t*-tests were conducted on the participants’ accuracy for each level of consistency. The results revealed that the participants’ recognition accuracy in all the three consistency conditions was significantly higher than the chance level (*t*_(24)_ = 16.83, *p* < 0.001, *t*_(24)_ = 5.43, *p* < 0.001, *t*_(24)_ = 4.66, *p* < 0.001), for high-, moderate-, and low-consistency levels, respectively. These results suggest that participants can acquire positional regularities of radicals through statistical learning.

A repeated measures ANOVA with the experimental condition as within factor was also performed on the accuracy rate and reaction time for the recognition test to further examine the participants’ sensitivity to the consistency of radicals. For the accuracy, the results showed that there was a significant consistency effect (*F*_(2,48)_ = 32.30, *p* < 0.001, *η*^2^ = 0.57). Follow-up contrasts demonstrated that participants were more accurate in the high-consistency condition than the moderate- and low-consistency conditions (*p*s < 0.001). It suggests that the participants were sensitive to the positional consistency of target radicals, and the high consistency facilitated their recognition of pseudocharacters. However, there was no significant difference among all three conditions on the reaction time (*F*_(2,48)_ = 0.77, *p* = 0.43, *η*^2^ = 0.03).

### ERP Data

**Figure 2:**
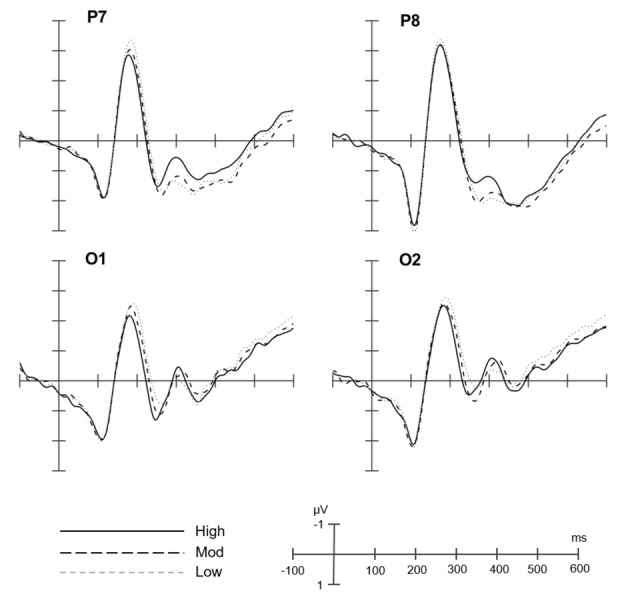
Grand averaged event-related potential (ERP) waveforms of low-, moderate-, and high-consistency conditions in P1, O1, P8, and O2 electrodes. High, high-consistency level; Mod, moderate-consistency level; Low, low-consistency level.

**Figure 3:**
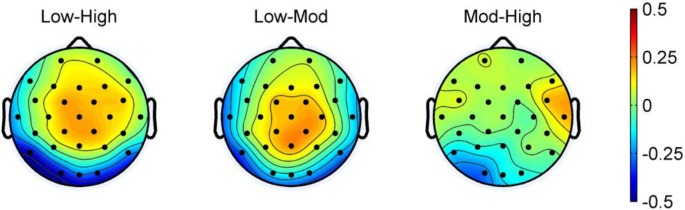
Topographic maps of consistency effects. High, high-consistency level; Mod, moderate-consistency level; Low, low-consistency level.

Figure 2 shows the grand average of ERPs at the four selected electrodes for all conditions. Figure 3 shows the topographic maps of the N1 for the difference between high consistency vs. low consistency, high consistency vs. moderate consistency, and moderate consistency vs. low consistency. The mean amplitude at each selected electrode is shown in Table 2.

**Table 2:** Mean amplitudes between 150 and 210 ms for each condition in the ERP learning task at the electrodes of P7, O1, P8, and O2.

Conditions	P7	O1	P8	O2
Low consistency	−2.681 (2.768)	−2.056 (2.825)	−2.787 (2.944)	−2.314 (2.996)
Moderate consistency	−2.412 (2.813)	−1.914 (2.804)	−2.580 (2.967)	−2.124 (3.027)
High consistency	−2.241 (2.770)	−1.645 (2.714)	−2.558 (2.941)	−2.018 (3.003)

Repeated measures ANOVAs, with consistency level (low, moderate, and high), electrode (P, O), and laterality (left, right) as within-subject factors, revealed that the consistency effect was significant (*F*_(2,48)_ = 6.30, *p* < 0.01, *η*^2^ = 0.21). The follow-up comparisons showed that the mean amplitude for the low-consistency level (*M* = −2.46 μV) was more negative than for the high-consistency level (*M* = −2.12 μV); however, the difference between moderate-consistency level (*M* = −2.26 μV) and high-consistency level was not significant, and the difference between moderate-consistency level and low-consistency level was not significant. The effect of electrode was significant (*F*_(1,24)_ = 5.21, *p* < 0.05, *η*^2^ = 0.18). The mean amplitude for P7/8 was more negative than for O1/2. The effect of laterality was not significant (*F*_(1,24)_ = 0.44, *p* = 0.51, *η*^2^ = 0.02). The interaction of laterality by consistency level was not significant (*F*_(2,48)_ = 1.76, *p* = 0.18, *η*^2^ = 0.07). The interaction of electrode by consistency level was not significant (*F*_(2,48)_ = 0.86, *p* = 0.43, *η*^2^ = 0.04). The interaction of laterality by electrode was not significant (*F*_(1,24)_ = 0.07, *p* = 0.79, *η*^2^ = 0.00). Moreover, the three-way interaction of laterality by electrode by consistency level was not significant (*F*_(2,48)_ = 0.58, *p* = 0.57, *η*^2^ = 0.02).

## Discussion

Statistical learning is useful for acquiring orthographic regularities, such as spelling patterns, in alphabetic languages (e.g., Pacton et al., 2005; Treiman et al., 2018) and positional regularities in non-alphabetic Chinese (e.g., He and Tong, 2017). Our study is the first to investigate the time course and neural character correlate of statistical learning of one key aspect of Chinese orthographic regularities (i.e., positional regularities). Our core finding is that the low- and moderate-consistency levels elicited a larger neural response in the time window of 150–210 ms (i.e., the N1 component) than the high-consistency level over the occipital–temporal area of the brain (in particular, in the O1 electrode). This result suggests that the N1 component may be a possible neural indicator associated with statistical learning of positional regularities of Chinese character orthography and that statistical learning occurs in the early time window before 210 ms.

Consistent with previous behavioral studies (e.g., He and Tong, 2017), our results showed that all participants were able to acquire positional regularities through statistical learning and that consistency level impacted statistical learning performance, with better performance appearing for high rather than moderate- and low-consistency conditions. These results suggest that statistical learning is a potential mechanism underlying orthographic learning. Additionally, our results revealed that the neural response was more negative for the moderate- and low-consistency levels than for the high-consistency level in the time window of 150–210 ms, indicating that statistical learning of positional regularity may occur in the time window of the N1 component. Indeed, this finding aligns with previous ERP studies showing that the N1 component is related to orthographic processing across writing systems (e.g., Bentin et al., 1999; Maurer et al., 2006, 2008; Zhao et al., 2012) and learning processes (e.g., McCandliss et al., 1997).

Our findings may be understood in terms of two competing hypotheses concerning the underlying mechanisms of the N1 effect. One assumes that the N1 effect was influenced by familiarity of the stimulus (e.g., Posner and McCandliss, 2000; Maurer et al., 2008). For example, a larger N1 was found for word and word-like stimuli than for visual controls, and for real word than for pseudowords in both children and adults (e.g., Maurer et al., 2005, 2006).

In contrast, the other hypothesis argues that the N1 effect was modulated by orthographic regularity but not by the familiarity of letter strings (McCandliss et al., 1997). For example, in a study by McCandliss et al. (1997), participants were trained to learn 60 Keki words. Their brain responses to four types of stimuli, i.e., familiar English words, Keki words, Keki control words (i.e., unfamiliar words), and English consonant strings, were recorded before training, 10 days after training, and 5 weeks after training. The ERP results across all three sessions showed that the familiar English stimuli elicited the least negative N1, while the unfamiliar English consonant strings elicited the most negative N1; the learned Keki and Keki-control strings were in between. The authors thus argued that the negativity level of the N1 is associated with the degree of orthographic regularity of the letter string, with greater orthographic regularity associated with less negative N1 response.

In line with the orthographic regularity hypothesis of the N1 effect, we found that the low-consistency level (60% or irregular: the target radicals occur in several different positions) elicited the most negative N1 response, followed by the moderate-consistency level (80% or semi-regular: the target radicals have a primary and secondary positions), with the high-consistency level (100% or regular: the target radicals always appear in one specific position) eliciting the least negative N1 response. Additionally, the topographic distribution showed that the N1 was located in the left occipital–temporal areas, particularly, in the O1 electrode, which is also consistent with previous ERP and brain imagining studies showing posterior areas related to visual orthography (e.g., McCandliss et al., 2003; Maurer et al., 2005, 2008). Taken together, our findings are in accordance with the hypothesis that the N1 is influenced by orthographic regularity and also suggest that the N1 could be a neural indicator of statistical learning of positional regularity in Chinese.

It is noted that the pseudocharacters used in this study were created using real Geba characters that were used in the Naxi minority in Western China (Li, 2014), but which have never been exposed or taught to the participants. It is suspected that the results of the present study could be different from the results using real Chinese characters. In fact, a recent behavioral study (He, 2015) has examined this question using different visual learning materials including Chinese pseudocharacters (e.g., 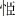, 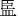), Geba pseudocharacters (e.g., 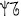, 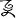), and nameless figures (e.g., 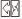, 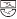). The results from the recognition test suggested that the participants could learn the positional configurations of stimuli from statistical learning with all types of orthographic codes, although the difference of orthographic types did influence the participants’ accuracy and reaction time in recognizing the exposed stimuli. Nevertheless, researchers should further clarify the answer to this question by directly comparing how different visual materials influence the neural mechanism of orthographic statistical learning in Chinese.

By employing an artificial orthographic learning paradigm to systematically manipulate statistical consistency of the target radicals, the present study is the first to demonstrate that the orthographic statistical learning process occurs quite early within the time window of 150–210 ms and that the N1 might be a neural indicator of orthographic statistical learning in Chinese. With the statistical learning of positional regularity ERP component (N1) identified, it would be worthwhile for future research to investigate whether the same component is evoked in other aspects of statistical learning of Chinese character orthography, such as orthography–phonology and orthography–semantics mappings.

## Data Availability Statement

The datasets generated for this study are available on request to the corresponding author.

## Ethics Statement

The studies involving human participants were reviewed and approved by Hangzhou Normal University. The patients/participants provided their written informed consent to participate in this study.

## Author Contributions

XT and ST conceived and designed the experiment. YW collected the data. XT and YW analyzed the data. XT and ST wrote the article.

## Conflict of Interest

The authors declare that the research was conducted in the absence of any commercial or financial relationships that could be construed as a potential conflict of interest.
